# Pediatrician’s role on patients with learning disabilities: a pilot study

**DOI:** 10.1590/1984-0462/2025/43/2024106

**Published:** 2024-11-29

**Authors:** Vanessa Pacini Inaba Fernandes

**Affiliations:** a Prefeitura Municipal de Sumaré – Sumaré (SP), Brazil.

**Keywords:** Learning disability, Screening test, Child, Literacy, Deficiências de aprendizagem, Detecção precoce, Criança, Alfabetização

## Abstract

**Objective:**

Learning disability leads to school dropout and low self-esteem in childhood, low socioeconomic status, increased criminality, and incarceration in adulthood. Pediatricians are key professionals who can diagnose and prevent this. The objective of the study was the early detection and reference of children with learning disabilities as identified by their schoolteachers.

**Methods:**

The protocol included: specific anamnesis with parents; Snellen test; audiometry and central auditory processing test; referral to speech therapists, ophthalmologists, otorhinolaryngologists, psychiatrists, psychologists, and neurologists if necessary; validated screening tests to evaluate literacy and mathematics reasoning skills; and development of a report for parents and teachers on the suspected diagnosis, professional referral, and curricular adjustments.

**Results:**

A total of 15 patients were evaluated in 1 year, with a mean age of 10.3 years, median school fourth grade, and mostly males (80%). The time for final report delivery was 6.4 months. Visual impairment was identified in 35.7% and central auditory processing impairment in 100% of patients. For writing and reading skills, seven children had below average and two children had average scores; for mathematics skills, five had below average, one average, and one higher score. Six children were illiterate and were suspected of having autism spectrum disorder, attention-deficit hyperactivity disorder, oppositional defiant disorder, intelligence disability, or borderline intelligence coefficient.

**Conclusions:**

A specific protocol helped to identify sensory organ impairments and psychological and psychiatric conditions, quantify school hardship, and provide a report with a suspected diagnosis and referral for treatment of learning disabilities. Larger population studies and a control group are necessary to validate this protocol.

## RESUMO

**Objetivo:**

A dificuldade de aprendizagem é causa de abandono escolar, redução de renda e risco de encarceramento. O pediatra é um profissional-chave para o diagnóstico e prevenção desse desfecho. O objetivo deste estudo foi detectar precocemente e referir crianças com dificuldade de aprendizagem encaminhadas da escola.

**Métodos:**

O protocolo incluía a realização de anamnese com os pais; teste de Snellen, audiometria e processamento auditivo central; encaminhamento para fonoaudiólogo, oftalmologista, otorrinolaringologista, psiquiatra, psicólogo e neurologista se necessário; teste de rastreio validado para leitura, escrita e matemática; relatório final com a suspeita diagnóstica, encaminhamento para profissionais e adequação curricular.

**Resultados:**

Em um ano foram avaliados 15 pacientes, com média de idade 10,3 anos, 80% do sexo masculino e mediana de série escolar quarto ano. O relatório foi entregue para pais em média após 6,4 meses. Alterações visuais e de processamento auditivo foram observadas em 35,7 e 100%, respectivamente. O resultado para escrita e leitura foi abaixo da média em sete crianças e na média em duas; para matemática, cinco crianças ficaram abaixo da média, uma na média e uma acima da média. Seis crianças não eram alfabetizadas e as suspeitas diagnósticas foram transtorno do espectro autista, déficit de atenção e hiperatividade, transtorno desafiador opositivo, deficiência intelectual ou inteligência *borderline*.

**Conclusões:**

O protocolo ajudou a identificar causas orgânicas de dificuldade de aprendizagem, quantificou a dificuldade e produziu um relatório com suspeita diagnóstica e encaminhamentos necessários para auxiliar no tratamento das crianças. Estudos com maiores populações e grupo controle são necessários para validar o protocolo.

## INTRODUCTION

Learning disability is a frequent, permanent, and intrinsic condition, with a reduction in oral language skills and consequently in reading and writing.^1^ The diagnosis is clinical, and treatment involves outpatient sessions with specialized psychologists, speech therapists, and educators for at least 6 months after complete literacy to distinguish between simple school struggle versus learning disabilities with diminished skills in reading (dyslexia), writing (dysorthographia), or mathematics (dyscalculia). This is distinct from ordinary school difficulty, as this condition is temporary and caused by external factors, like environmental, psychological, and educational problems.

The pediatrician’s role in identifying and referring these patients was demonstrated in a recent article in which authors showed that emotional and academic damage in patients with learning disabilities is a risk for professional failure, incarceration, and unemployment in adult life. They suggest that a huge effort from the government and institutions is needed to promote continuous training of pediatricians in diagnosing and intervening in patients with dyslexia.^2^ Pediatricians should use screening tests to identify children at risk of learning disabilities. Even though a variety of screening tests are available in English language, there is no standard test for pediatricians to use in their clinics.^3^ Moreover, only one test is validated for the Portuguese language (“Test Identification Signs of Dyslexia — TISD”), and it is only available for psychologists and speech therapists, not for pediatricians.^4^

When it comes to the public education system in Brazil, learning disabilities have two pathways for diagnosis: directly from the school to specialized learning disabilities centers or from basic public health professionals and referrals. Unfortunately, specialized learning disabilities centers are scarce, with long waiting lines. At the same time, public primary care center professionals, including pediatricians, lack awareness of learning disabilities for assertively identifying, referring, and treating children at risk.

During 15 years in primary health care in the city of Sumaré, a mid-sized urban city in Southeast Brazil with 279,545 inhabitants, at least eight patients with learning disabilities who were referred from school to be evaluated were recalled. In total, three dropped out of school after 12 years of age, two of them were illiterate; two only succeeded in being evaluated in a specialized center for learning disabilities, but were not treated; the last three returned to the primary health facility after COVID-19 pandemic without any professional evaluation. The lack of resolutive follow-up on these patients showed the urgency of developing a protocol to evaluate these and future children with learning disabilities. Therefore, the goal of this pilot study was to develop a protocol for investigating learning disabilities, which could be reproduced by other primary care pediatricians for additional validation in order to promote early diagnosis and adequate referral to professionals able to further evaluate and treat these patients.

## METHOD

This is a non-randomized clinical pilot trial in a group of children with suspected learning disabilities without prior diagnosis, referred from school for evaluation at a specific primary healthcare facility (Sao Judas Tadeu). A single pediatrician attended these patients, and after the consultation, they were enrolled in the study. All participants (parents and children) of the study agreed to participate and filled out the informed consent approved by the local Ethics Committee (protocol number 75480923.7.0000.0167). The study was performed for a year.

Inclusion criteria involved children with learning disabilities from first to ninth grade with a referral from school. Children without school referral, with a definitive diagnosis of mental deficiency, autism spectrum disorder, attention-deficit hyperactivity disorder, and psychiatric conditions, were excluded from the study. After filling out the informed consent, four appointments were scheduled:

1. First appointment: anamnesis with parents in a 30-min visit in which information about pregnancy, delivery, neuropsychological development, familiar history of learning disability or mental disease, normal routine, and scholarship history were obtained. Also, at this appointment, a Snellen test was performed, and patients were referred for audiometry and a central auditory processing test.
2. Second appointment: a validated screening test with a 1-h duration to evaluate literacy and mathematics skills from first to ninth grade was performed. For first to second grade, “*Protocolo cognitivo-linguistico para fases iniciais*” *(PCL fase iniciais)* ^5^ individual version was used; for third to fifth grade, “*Protocolo cognitivo-linguistico revisado*” (PCL-R)^6^ individual version was applied; for sixth to ninth grades, “*Teste de desempenho escolar-2*” (TDE II)^7^ was used.
3. Third appointment: a 40-min session was used for the collective version of PCL *fases iniciais* and PCL-R or Binet–Simon intelligence coefficient test,^8^ if necessary. The last test was applied in children with very low punctuation on PCL-R (2 points for average or higher scores) or in illiterate patients by third grade.
4. Fourth appointment: a 20-min appointment with parents for reading of the report with a diagnostic hypothesis and referral to specialized professionals if necessary; patients were referred to the ophthalmologist if an altered Snellen test was identified; to the otorhinolaryngologist if there was an altered audiometry; to perform games on a specific fare-paying platform (www.afinandocerebro.com.br) if central auditory processing was impaired; to speech therapy if there was a phonological impairment; to the psychiatrist or psychologist if a psychiatric or psychological condition was detected; and to the neurologist if cognitive impairment was suspected.

“PCL *fases iniciais*” and “PCL-R” are both open tests that can be applied by any health and education professional. They have two parts: a collective and an individual test. According to the scores obtained, each ability is classified on a lower, average, and higher result for each specific grade. If an individual test score is under 50% of the average, the child is considered at risk for learning disabilities. Skills evaluated in both tests include alphabet and name writing; copy of shapes; word, pseudoword, and picture dictation; words and pseudowords reading; word, pseudoword, and number reverse sequence repetition; skills predictors of reading; rapid automatized naming of figures and numbers; visual memory of shapes; number sequence dictation (former); and mathematics calculation (last).

“TDE II” is also an open test with two distinguished protocols, one from the first to fourth or fifth grade and the second from fifth or sixth to ninth grades, and evaluates writing skills, mathematics reasoning, and reading, in this sequence. In the end, scores for each subject are compared in a chart with percentiles and classified as lower, average, and higher for each school grade. It is a broad-spectrum test but cannot be used in illiterate children.

Finally, the Binet-Simon intelligence coefficient test is a traditional open test that has a six-question quiz for each age from 3 to 16 years old, with two points per question. If the child can solve all six questions for her age, questions of a superior age are offered until she fails. On the other hand, if the child can’t solve the question for her age, questions of inferior age are offered until she can solve it. All points over and under chronological age tests are counted and multiplied by 12 (mental age), and the intelligence coefficient is calculated as the result of the division of mental age by chronological age (age multiplied by 12 plus months of age). The normal intelligence coefficient for the population is between 85 and 115,^9^ and intelligence deficiency is considered under 70.^10^

For sample size calculation, a study with second-grade students that evaluated a risk group with learning disabilities and a control group with “PCL *fases iniciais*” was used.^11^ Based on this study, a sample size of 15 children was defined, as children with learning disabilities referred to primary health care are scarcer than when evaluated directly in a school environment. Mean, median, and standard deviation were used for data analysis. The reason for not having a control group is that at the beginning of the study, three patients (20% of sample size estimation) that were evaluated before the COVID-19 pandemic returned in 2022 with no screening or treatment. The authors considered it unethical to not offer to these patients and to the incoming patients the new screening option, given the failure of the previous traditional screening and referral methods.

## RESULTS

From September 2022 to December 2023, 15 children were evaluated. The mean age of the patients was 10.3 years (range: 7.6–14.9 years), mostly male (80%). The median school grade was fourth grade. Patients waited a mean of 6.4 months (range: 2–11 months) to receive the final report, due to a long waiting list and reduced scheduling slots with the single pediatrician.

Positive learning disability family history was identified in 13 patients (86.6%), 5 had positive psychiatric disease family history (33.3%) and 10 had environmental problems (66.6%), mostly parents’ divorce and death of a parent or a close relative. Most of the children had personal problems (80%), including prematurity, small for gestational age, suspicions of psychiatric conditions, non-infectious microcephaly, treated congenital neurosyphilis, clinical hypothyroidism, shyness, inattention, and blunted affection.

In total, five children needed refractive error correction (35.7%, n=14), all had normal audiometry (100%, n=12), and at least three disabilities on the central auditory processing test (100%, n=10). Notably, three patients were referred to a neurologist (20%) for mental deficiency investigation because of very poor PCL-R test performance (two abilities with average or higher results of 16) and illiteracy. The Binet–Simon coefficient index for these patients was 63 (mild intellectual disability), 77, and 78 (borderline intelligence coefficient). In total, four patients were referred to the psychiatrist (26.6%) and five to the psychologist (33.3%) for investigation of autism spectrum disorder, attention-deficit hyperactivity disorder, anxiety, and oppositional defiant disorder.

Notably, four patients were from first and second grades and underwent a “PCL *fases iniciais*” individual test, with mean scores for average and higher results 10.2 (10–11 of 14 abilities, n=4). After that, literate patients underwent a collective test, and mean scores for average and higher results were 4.5 (four to five of seven abilities, n=2). Although patients scored over 50% of average and higher in this test, most of them were for skills predictors of reading. For reading and writing skills, most of these patients scored below the average. Notably, two patients from this group were considered illiterate.

In total, eight patients were from third to fifth grades and underwent the “PCL-R” individual test. Mean scores for average and higher results were 3.8 (2–7 of 16 skills, n=8). After that, literate patients (scores 5–7) underwent a collective test, and mean scores for average and higher results were 3.75 (3–4 of 7 skills, n=4). These patients scored below average in reading, mathematics, and writing skills, except one patient with a result in mathematics above the average. Notably, four patients in the group were illiterate. Patient results for “PCL *fases iniciais*” and “PCL-R” are shown in Table 1.

**Table 1:** Results of “*Protocolo cognitivo-linguistico fases iniciais*” and “*Protocolo cognitivo-linguistico revisado*”.

	n	Mean	Range	Number of skills
“PCL fases iniciais” individual test Superior and average results	4	10.2	10–11	14
“PCL fases iniciais” collective test Superior and average results	2	4.5	4–5	7
“PCL-R” individual test Superior and average results	8	3.8	2–7	16
“PCL-R” collective test Superior and average results	4	3.75	3–4	7

PCL: *Protocolo cognitivo-linguistico;* PCL-R: *Protocolo cognitivo-linguistico revisado.*

Three patients were from sixth to ninth grades and underwent a “TDE II” evaluation. “TDE II” writing, mathematics, and reading efficiency test results were, on average, 66.6, 33.3, and 66.6%. The results were below average at 33.3, 66.6, and 33.3%, respectively. Table 2 shows the results of “TDE II” tests. Finally, we could observe a total of six illiterate patients.

**Table 2:** “*Teste de desempenho escolar-2*” test results and reading efficiency for literate patients.

	Number	Higher	Average (%)	Lower (%)
TDE II writing efficiency	3	0	66.6	33.3
TDE II mathematics efficiency	3	0	33.3	66.6
TDE II reading efficiency	3	0	66.6	33.3

TDE II: *Teste de desempenho escolar-2.*

A summary of the skills’ evaluation of those who could read was: for writing and reading skills, seven children were below average and two had an average score; for mathematics skills, five were below average, one average, and one had a score above average. Screening tests only evaluated mathematics skills in children over third grade. Table 3 shows a summary of the results for writing, reading, and mathematics skills evaluated in all three tests.

**Table 3:** All patients’ results for writing, reading, and mathematics skills.

Skill	Higher score	Average score	Lower score
Writing (n=9)	0	2	7
Reading (n=9)	0	2	7
Mathematics (n=7)	1	1	5
Illiterate	6 patients		

For the final report, the diagnostic hypotheses were school difficulty or learning disorder in nine children (60%), two with autism spectrum disorder (13.3%), two with borderline intelligence coefficient (13.3%), one with intelligence disability (6.6%), one with attention-deficit and hyperactivity disorder (6.6%), and one with oppositional defiant disorder (6.6%). All hypotheses were not final, and the children were referred to specialists for confirmation (psychiatrist, neurologist, psychologist, speech therapist, and educational psychologist).

## DISCUSSION

Illiteracy is a significant problem in Brazil, affecting around 11 million people in 2019^12^ with almost 50 million people between 14- and 29 years old not having graduated from high school (20.2%). Furthermore, the quality of Brazilian education shown in the Program for International Student Assessment (PISA)^13^ is below the world average score since 2000 in reading, mathematics, and sciences. High school dropout and low quality of education reduce job opportunities and the economic status of the youth, leading to an increase in criminality. A 2016 study on the Brazilian prison population showed that 75% of prisoners were 18–34 years old, and 67% had dropped out of school before their ninth year.^14^ In addition to this context, the COVID-19 pandemic also reduced academic achievement, as Brazil had 78 weeks of full or partial school closure, and some public schools only opened in 2021.^15,16^

This environment shows the importance of identifying children struggling in initial school grades to help them achieve their full potential. Our patients’ clinical characteristics were in agreement with previous literature: most patients were male (80%), with a positive family history of learning disabilities (86.6%), environmental difficulties (66.6%), and comorbidities associated in almost 30% of patients.^2^ There were sensory disabilities, like refractive visual errors (35.7%) and impaired central auditory processing (100%). The mean time to receive a final report was around 6 months, but as queues were reduced, the last child received his final report after only 2 months of evaluation. Unfortunately, children showed up for primary health care evaluation very late, at a mean age of 10.3 years, by fourth grade, sadly in agreement with Brazilian statistics.^17^ If children had shown up earlier, the evaluation would be simpler by simple screening tests, such as “PCL *fases iniciais*” in elementary years, which evaluate literacy learning skills that can predict reading comprehension by the first year of high school (tenth grade).^18^ As shown in the results, most studied children were less than average for reading (86.6%), writing (86.6%), and mathematics skills (84.6%) and needed urgent diagnosis and treatment of the condition.

Regarding our small sample size, this was justified by a reduced number of referrals from schools, as only 10 children in 2022, 5 children in 2023, and 4 children in 2024 (not included in the study) showed up in the clinical appointments with a school report. As dyslexia world prevalence is between 3 and 17%,^19^ and we have approximately 1300 pediatric clinical appointments a year, 5% of these appointments would result in 65 patients with dyslexia. Half of these patients are probably in state schools (the other half from municipal schools) that do not have access to specialized learning disabilities centers and would be referred from the school to a pediatric clinical appointment. Therefore, the small sample size and the lack of a control group may reduce the relevance of this study. However, as a model for reproduction by other pediatricians in a larger study with a control group, the results of this intervention may be validated and generalized in the future. The lack of a control group also prevented more advanced statistical analysis.

As described in the results, some children could not take both tests (collective and individual) for “PCL-R” and “PCL *fases iniciais*.” That happened in children who could not write, so they were unable to take dictates. Exposure of these children to something they were incapable of performing was unnecessary and harmful. In patients with lower scores, an intelligence coefficient test was taken to discard intelligence disability. All children accomplished all the tests they were asked to undergo.

This protocol is specific to the Brazilian population, as there is no validated screening test for dyslexia in Portuguese language that could be applied by pediatricians. All screening tests used were national. In general, speech therapists and psychologists apply the screening tests, and they take up to 5–6-h sessions for a whole evaluation (based on experts’ information). The performance of this protocol needed fewer hours to be accomplished (up to 3 h work) and also evaluated sensory impairments, child’s behavior, and included referral to specialists when needed, resulting in a more complete and time-effective evaluation. Costs for performing this protocol were not excessive, approximately US$417.00 for education training and materials for pediatricians.

In Brazil, pediatricians are still doing the traditional screening and referral method,^20,21^ but around the world, the application of effective protocols is starting to be used, despite the lack of standardization. Most are performed by speech therapists and psychologists, usually in schools rather than clinics. Two recent protocols were developed by pediatricians. The first one was made by a group of behavior pediatricians that investigated 41 children from 6 to 14 years of age attending the developmental-behavioral pediatrics clinic with a tool to evaluate reading. The tool was the Rapid Online Assessment of Reading (ROAR), which is an objective, gamified assessment that children take in a web browser without adult supervision. There was a positive correlation between ROAR standard low scores and basic reading skills <90, which suggests poor readers.^22^ A second study used The Reading House (TRH), a children’s book-based screener of emergent literacy skills in preschool-aged children. In total, 70 healthy children from 3 to 5 years old attending a pediatric center participated in the study.^23^ TRH and the scales Expressive Vocabulary Test, Second Edition (EVT-2), Comprehensive Test of Phonological Processing, Second Edition (CTOPP-2), Get Ready to Read! (GRTR), and the Pre-Reading Inventory of Phonological Awareness (PIPA) were applied before MRI of the brain. TRH scores correlated positively with other scales and greater thickness in left-sided language and visual cortical areas associated with reading activity.

In conclusion, a specific protocol for pediatricians would help identify sensory organ impairments and psychological and psychiatric conditions and quantify school difficulty to provide a professional assessment for children for a definitive diagnosis and treatment. This is a low-cost, easy-to-perform protocol, with no special infrastructure requirements, depending only on the government and institutions promoting continuous training for pediatricians and providing specific materials. Studies with larger populations and a control group are necessary to validate this protocol for clinical use by Brazilian pediatricians.

## Data Availability

The database that originated the article is available with the corresponding author. CAAE: 75480923.7.0000.0167
